# Resident and informal caregiver involvement in medication-related decision-making and the medicines’ pathway in nursing homes: experiences and perceived opportunities of healthcare professionals

**DOI:** 10.1186/s12877-022-02773-6

**Published:** 2022-01-27

**Authors:** Amber Damiaens, Ann Van Hecke, Jan De Lepeleire, Veerle Foulon

**Affiliations:** 1 Clinical Pharmacology and Pharmacotherapy, Department of Pharmaceutical and Pharmacological Sciences, KU Leuven, O&N II - Herestraat 49 - Box 521, B-3000 Leuven, Belgium; 2 Department of Nursing director, Ghent University Hospital, University Centre for Nursing and Midwifery, Department of Public Health and Primary Care, UGent, Corneel Heymanslaan 10, B- 9000 Ghent, Belgium; 3 Academic Center for General Practice, Department of Public Health and Primary Care, KU Leuven, Kapucijnenvoer 7 - Blok H - Box 7001, B-3000 Leuven, Belgium

**Keywords:** Person-centered care, Pharmacotherapy, Nursing home residents, Informal caregivers, Qualitative study

## Abstract

**Background:**

Person-centered care has been shown to be beneficial for nursing home residents. The know-how and attitude of healthcare professionals, however, can make its implementation difficult. Also, research on person-centered care with regard to medication decision-making and the medicines’ pathway in nursing homes is lacking. This study aimed to provide an understanding of healthcare professionals’ attitudes and perspectives on current resident and informal caregiver involvement in medication decision-making and the medicines’ pathway in nursing homes.

**Methods:**

A qualitative, explorative study using semi-structured interviews with a sample of 25 healthcare professionals from four different nursing homes was performed. Interview transcripts were analyzed by means of an inductive thematic framework.

**Results:**

Three overarching domains were identified: 1) features of, 2) drivers and barriers for, and 3) perceived consequences of resident and informal caregiver involvement in medication decision-making and the medicines’ pathway. Involvement was mainly initiated by residents and informal caregivers themselves, pointing towards information and participation needs among both groups. Nevertheless, actions of healthcare professionals towards resident and informal caregiver involvement were mainly reactive and fragmentary. Their actions were influenced by the perception of residents and informal caregivers’ desire and capabilities to be involved, the perception of their own professional role, but also by organizational factors such as the nursing home’s philosophy. Furthermore, organizational concerns tempered the motivation to provide residents and informal caregivers with more medication-related responsibilities.

**Conclusions:**

Resident and informal caregiver involvement in medication decision-making and the medicines’ pathway remains limited in nursing homes. Information and participation needs of residents and informal caregivers were not fully acknowledged by healthcare professionals. As such, we can conclude that there is a need for initiatives, both on an individual and on an organizational level, to create and improve awareness on opportunities to improve resident and informal caregiver involvement in medication decision-making and the medicines’ pathway.

## Background

Care that is guided by an individual’s preferences and aims is referred to as person-centered care or PCC [1]. It has been shown to be beneficial for older patients, including nursing home residents (NHRs). A systematic review by Brownie et al. showed that PCC can lead to an improvement in perceived QoL, reduction in feelings such as boredom and depression and better communication between nursing home staff and NHRs [2]. Likewise, NHRs who are engaged in setting goals for care are more likely to improve in physical and mental well-being [3–5]. Furthermore, residents’ autonomy has been shown to be strongly related to their well-being [6].

The realization and provision of PCC, however, remains challenging. Important barriers are the know-how and attitude of healthcare professionals [7]. Healthcare professionals (HCPs) struggle to find the right approach to reconcile their own and residents’ priorities, have strong opinions themselves about what is best for the NHR, and are often unaware of residents’ involvement preferences [8–10]. Furthermore, HCPs often perceive NHRs not wanting to be involved, nor being capable to be involved in their care [11]. The need among HCPs to be in control might also hinder involving residents and informal caregivers [12]. Residents, on the other hand, might have the impression their HCPs are not receptive to their questions and requested services [13].

Besides challenges in the provision of PCC in general, research on PCC with regard to medication-related activities and treatment decisions, especially in nursing homes (NHs), is scarce. The majority of research on medicines’ optimization interventions in NHs is person-centered in its aim (i.e. improving the resident’s medical treatment), but not in its approach. Residents’ and informal caregivers’ involvement in medication-related decision-making processes remains limited or absent [6, 14–17] and outcomes are usually measured at the medicines level (e.g. appropriateness of prescribing) but rarely on the resident’s level (e.g. QoL) [18]. Moreover, residents hardly maintain any autonomy or responsibilities in their medicines’ pathway in the NH (e.g. prescribing, decision-making, purchasing, storing, administering, … of medication) [19], while prior to admission they may have self-organized their medicines, with or without any help from an informal caregiver [15, 20, 21]. In an attempt to support this independence and autonomy, some countries (e.g. Australia, UK) provide guidelines on the self-management of medication (i.e. storage and administration) in residential care facilities, including a risk and competency assessment of the resident [22, 23]. In Belgium, however, no such guidelines exist, and in most cases the resident or the informal caregiver is asked at admission to sign an agreement by which they consent to all medication-related responsibilities being handled by the NH staff.

The RESPECT-study (RESident’s Participation in the Evaluation and Customization of Therapy), that was recently set up in Belgium, aims to explore opportunities for resident and informal caregiver involvement in medication decision-making and the medicines’ pathway in nursing homes.

To inform person-centered practices and tackle healthcare professional-related barriers, a thorough understanding of current medication-related practices and the views and attitudes of HCPs involved in the care of NHRs on resident and informal caregiver involvement, is crucial. Therefore, the current study aimed to answer the following research questions:

- How do HCPs currently involve NHRs and informal caregivers in medication decision-making and in the medicines’ pathway?
- What are the underlying motives of HCPs in their current initiatives towards involving residents and informal caregivers in medication decision-making and in the medicines’ pathway?
- What are, according to HCPs, (additional) opportunities for involvement of NHRs and informal caregivers in medication decision-making and in the medicines’ pathway?
- What are, according to HCPs, the benefits and consequences of involvement of NHRs and informal caregivers in medication decision-making and in the medicines’ pathway?

## Methods

### Design

A qualitative, explorative study was executed by means of semi-structured interviews, across Flanders, the Flemish-speaking part of Belgium, and Brussels.

### Participants

A list of NHs in Belgium, available through the website of the National Institute for Health and Disability Insurance was used (2018) for the selection of NHs. Three criteria were used to purposively invite NHs for participation: ownership status (i.e. private nonprofit, private for-profit or public), number of beds (i.e. 35 to 80 beds, 81 to 150 beds, and more than 150 beds) and location (i.e. one in each Flemish province and one in Brussels). A total of 26 NHs was invited by e-mail and telephone. When interested to participate, additional information was provided through mail and phone. Seven NHs agreed to participate. Three of these NHs were excluded from the final sample because of the unexpected resignation of a head nurse, because of being unreachable at the time of the interviews or because another NH was already included for that location criterium. This resulted in a final set of four participating NHs (see Table 1). An additional characteristic ‘small-scale, homelike facility’ was added to describe the sample of NHs. For each NH, a local study liaison was appointed who acted as the contact point between the research team and the NH and facilitated the recruitment of participants.

**Table 1:** Characteristics of participating NHs

NH	Ownership status	Total number of beds	Small-scale, homelike?	Location (region)
1	Private nonprofit	112	Yes	Flemish Brabant (Flanders)
2	Private nonprofit	153	Yes	Limburg (Flanders)
3	Public	180	No	West Flanders (Flanders)
4	Private nonprofit	85	No	Brussels (Brussels Capital Region)

Purposive sampling was applied to recruit HCPs of the participating NHs for the interviews. Inclusion criteria were Dutch or French speaking and being actively involved as a HCP in the medicines’ pathway of the NH. All HCPs involved in the medicines’ pathway (i.e. GPs, pharmacists, nurses and care aids) were considered for participation in this study. In Belgium, GPs maintain the responsibility over the resident’s medication use. Residents remain free to choose their GP, resulting in a large number of visiting GPs for each NH. Furthermore, for each NH, one GP is appointed as a coordinating physician (CP), who is responsible for the therapeutic policy of the NH, including the medicines’ pathway. Medicines are provided by hospital or community pharmacies, as chosen by the NH itself. Monitoring of residents with regard to their medication is mainly performed by GPs, nurses, and care aids. Nurses and care aids are both involved in medication administration and are easily accessible points of contact for NHRs and informal caregivers for questions and remarks regarding the resident’s medication.

Each NH was asked to include at least the CP, the pharmacist and one head nurse for the interviews. Besides this, NHs were invited to suggest other HCPs involved in their medicines’ pathway. The aim was to include a variation of profiles of HCPs to ensure that all relevant HCPs were represented in the final sample. Staff active in the NH itself (e.g. nurses and care aids) was approached by the study liaison, who provided the research team with an overview of the availability of staff that agreed to participate. Upon receipt of this overview, the research team and study liaison agreed on one or more dates on which the interviews would be performed in the NH. Healthcare professionals who were not typically present in the NH (e.g. GPs and pharmacists) were contacted by the research team, after agreeing to be contacted and having their contact details provided to the research team by the study liaison. Consequently, a member of the research team contacted each of these HCPs individually to agree on a date and location for the interview.

### Data collection

Semi-structured interviews were performed between August 2019 and December 2020. Interview guide development was based on the composing activities of the medicines’ pathway, as identified by Strauven et al. [19]. The pathway consists of eight processes, going from admission of the resident over medication prescribing to medication administration and monitoring of medication (side-)effects. These processes are further divided into key activities, representing all medication-related tasks occurring in a NH. Open-ended questions were used to encourage participants to openly talk about their experiences and perspectives on resident and informal caregiver involvement regarding each activity of the medicines’ pathway, including medication decision-making. Examples of questions were ‘How does [activity of the medicines’ pathway] occur at the NH?’, ‘What role does the resident/informal caregiver play in [activity of the medicines’ pathway]?’, and ‘What role could residents/informal caregivers themselves take on in this [activity of the medicines’ pathway]?’. Whenever appropriate, additional open-ended questions were asked to further clarify the HCP’s response.

Interviews were performed by AD, AJ, PE, VF, a team consisting of both unexperienced and highly experienced researchers, each with a background in pharmacy. Interview locations included meeting rooms or (calm) communal living areas in the NH, as well as quiet locations outside of the NH (e.g. pharmacy).

All interviews were audio-recorded and subsequently transcribed ad verbatim. Transcripts were stored on a password-protected computer and were reviewed for accuracy while listening to the recordings.

Prior to or after finalization of each interview, a small self-reported questionnaire on demographic characteristics of the HCP was collected.

Data collection and analysis of the interviews was performed in an iterative process. Subsequently, as is common in qualitative research, the interview guide evolved into its final form over the course of the study. Themes identified in early interviews became probes for later interviews (e.g. experiences with NHR (re) admission, experiences with medication changes, information and participation needs of residents and informal caregivers, current resident and informal caregiver involvement initiatives, and opportunities for a person-centered medication review).

### Data analysis

Transcripts were analyzed by means of inductive thematic framework [24]. Analysis was performed by an interprofessional team, consisting of researchers with a background in pharmacy and nursing and with experience in topics such as patient participation and medication optimization interventions and qualitative research (AD, VF, and AVH). The researchers independently read the interview transcripts, highlighted meaningful paragraphs and noted preliminary reflective comments. Regular team discussions were held to develop (sub) themes, to endorse the identified (sub) themes and to ensure a rigorous and reliable data analysis.

Quotes were selected to illustrate the identified (sub)themes. Selection of these quotes and translation into English was performed by the first author (AD). Subsequently, quotes were checked for relevancy and accuracy by two independent members of the research team (VF and AVH).

### Rigor

Different approaches were applied to ensure rigor of the study. First, all study procedures were documented carefully. Second, several forms of triangulation were applied. Data analysis was performed by an interdisciplinary research team (see *‘Data analysis’*), establishing investigator triangulation. Also, by recruiting all relevant HCPs involved in the care of NHRs, data source triangulation was ensured. To further increase study rigor, the research team regularly held meetings to discuss findings (see *‘Data analysis’*). Moreover, this approach reduced researcher bias by improving researchers’ reflexivity.

### Ethical considerations

The study was approved by the Ethics Committee Research UZ/KU Leuven in May 2019 and was conducted in accordance with the principles of the Declaration of Helsinki. Written, informed consent for participation was collected prior to each interview, with the exception of two interviews that were performed via Zoom. In these cases, informed consent was collected through e-mail.

## Results

A total of 25 HCPs was interviewed, across a range of ages and years of experience with working in or for the NH (see Table 2). Interviews lasted 13 to 79 min, with an average of 50 min.

**Table 2:** Description of participants

Interviews, ***N***	25
Healthcare professional, *type (n)*	
Coordinating physician	4
General practitioner	3
Nurse	8
Pharmacist	3
Care aid	5
Director	2
Age, *n*	
≤ 25	1
26–35	8
36–45	3
46–55	8
> 55	5
Gender, *n*	
Female	18
Male	7
Number of years since graduation, *n*^*a*^	
≤ 5	2
6–15	11
16–25	2
> 25	9
Number of years actively working in or for the NH, *n*	
≤ 5	6
6–15	11
16–25	6
> 25	2

^a^one missing value

**Fig. 1:**
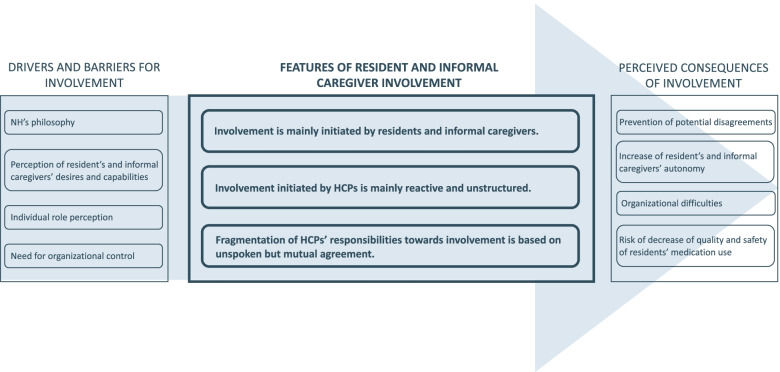
Drivers and barriers, features, and perceived consequences of resident and informal caregiver involvement in medication decision-making and in the medicines’ pathway, as perceived by HCPs

Themes that were derived from the interviews, could be grouped in three overarching domains: 1) features of resident and informal caregiver involvement, 2) drivers and barriers for resident and informal caregiver involvement, and 3) perceived consequences of resident and informal caregiver involvement in medication decision-making and the medicines pathway. The themes are described hereafter in an integrated manner, supported by illustrative quotes. An overview of themes and overarching domains is provided in Fig. 1.

### Involvement is mainly initiated by residents and informal caregivers

Healthcare professionals provided several examples of how residents and informal caregivers are currently involved in the medicines’ pathway. It became clear that several residents take up responsibility during medication administration rounds by exercising visual control on the (oral) medication they receive: they check the number and visual properties of tablets and consult a member of staff when potentially observing an error.

*“Every resident here knows perfectly how many tablets they’re having each morning because they always count them. ( …). Right now, for example, Asaflow [acetylsalicylic acid] is missing and will be changed into Asa100 [acetylsalicylic acid], so from next week onwards, a lot of residents will be asking ‘where is my little, round white tablet?’.”*

*Nurse 5, actively working in NH for 5 years*

Other examples include informal caregivers who request to purchase the resident’s medication at a pharmacy themselves (instead of having the NH staff order it), residents who ask to store (part of) their medication in their room, and residents who self-administer as well as informal caregivers who help with the administration of oral preparations. With regard to medication decision-making, HCPs mentioned that some residents and informal caregivers frequently ask questions as well as make suggestions about the resident’s medication. Besides this, most residents and informal caregivers inform staff about the resident’s symptoms or discomfort.

*“There are relatives who you never see, who are not really involved and there are others who specifically ask about it [the resident’s medication] or make an appointment at the practice to talk about it.”*

*GP 2, actively working in NH for 22 years*

Based on these examples, it could be stated that a feature of current resident and informal caregiver involvement is that both groups mainly initiate their involvement in medication decision-making and the medicines’ pathway themselves, and thus highly influence their own level of involvement therein (see Fig. 1, ‘Features of resident and informal caregiver involvement’).

### Involvement initiated by healthcare professionals is mainly reactive and unstructured

Healthcare professionals also provided examples of how they initiate resident and informal caregiver involvement in medication decision-making and the medicines’ pathway. These examples showed that their current actions are mostly related to informing residents and informal caregivers about medication changes. With regard to who and what to inform, however, actions varied among HCPs. Firstly, HCPs made a clear distinction between residents and informal caregivers who are found to be interested in medication-related topics and those who are not. Residents and informal caregivers perceived as interested were the ones who get informed more frequently in case something changes in the resident’s medication. Different cues indicate this interest to HCPs. A first one are questions and suggestions coming from both residents and informal caregivers, mostly related to the resident’s medication list and changes therein, or to changes observed on the pharmacy invoice. Some residents and informal caregivers even make concrete suggestions about the resident’s medication (e.g. to start or quit a specific medicine), which is clearly considered as ‘being interested’. Residents’ performance of visual control during medication administration rounds (cfr. supra), was found to be another cue of interest. With regard to informal caregivers, those who visit regularly and those who have previously expressed their disagreement on specific medication changes were known as informal caregivers interested in the resident’s condition and medication, respectively. Current actions of HCPs to involve residents and informal caregivers in medication decision-making and the medicines’ pathway could therefore be described as reactive (see Fig. 1, *‘Features of resident and informal caregiver involvement’*).

Besides their interest in medication-related topics, the condition of the resident was another important factor that influenced HCPs whether or not to inform residents. As an example, residents diagnosed with cognitive impairment (e.g. dementia) barely get notified about changes in their medication, since this was simply found to be not useful. However, it seemed that HCPs generally considered NHRs as ‘not capable’, also when talking about non-dementia residents. Based on the interviews, it was not clear how HCPs assessed a resident’s cognitive capacity in order to determine if the resident should be informed or not about a change in his/her medication.

*“If a resident is still cognitively capable, I tell them after examining him or her by saying: ‘you will be getting medication for that.’ As such, you tell them something beforehand and then afterwards you’ll make the prescription. That’s how it happens.”*

*GP 3, actively working for NH for 41 years*

Furthermore, HCPs more frequently inform residents and informal caregivers about medication changes that potentially result in observable side-effects (e.g. drowsiness), since this has previously caused disagreements with multiple residents and informal caregivers. While nurses named morphine preparations and antibiotics as examples, coordinating physicians and pharmacists more frequently spoke about psychotropic drugs. Also, medication changes resulting in a significant impact on the pharmacy invoice, raising awareness mostly from informal caregivers, were changes that residents and informal caregivers get informed about more often in order to prevent disputes. Subsequently, actions by HCPs to involve NHRs and informal caregivers in the medicines’ pathway are not only reactive, but also fragmentary and selective (see Fig. 1, *‘Features of resident and informal caregiver involvement’*).

*“Sometimes, when I'll increase someone’s drowsiness with a certain medicine, for example a neuroleptic, I discuss that with the family. Especially when the resident has never used that before.”*

*CP 2, actively working in NH for 9 years*

Nevertheless, during the interviews with HCPs within one nursing home, it became clear that systematically informing residents and informal caregivers is possible, as well as discussing potential medication changes with them prior to their implementation. These HCPs felt that this was mainly a result of the NH’s philosophy, which had a strong focus on providing holistic care, including resident and informal caregiver participation. Although this was also part of the philosophy of some of the other NHs, the difference seemed to be in the staff’s commitment towards making this philosophy tangible as well as keeping it alive through regular team discussions. The NH’s philosophy was therefore named as a potential driver for resident and informal caregiver involvement in medication decision-making and the medicines’ pathway (see Fig. 1, *‘Drivers and barriers for involvement’*).

### Fragmentation of healthcare professionals’ responsibilities towards involvement is based on unspoken but mutual agreement

The residents’ trust in their GP as well as the GP’s indispensable role in the prescribing process, made HCPs rely on this key figure for informing residents about medication changes. Furthermore, HCPs expect the GP to make the final decision about the requested involvement by residents (e.g. whether or not to allow them to store medication in their room).

*“When something is being changed [in the resident’s medication], they [the GPs] tell us but the GP should tell the resident as well. They go see the resident and then they come to us to discuss what [needs to be changed], a modification of a diuretic for example. They tell us but the resident doesn’t know anything about it in that way. Or we should tell them, but it makes a big difference if the GP tells them because for some [residents], the GP is holy.”*

*Nurse 1, actively working in NH for 11 years*

Coordinating physicians and GPs seemed to agree with the ‘principle’ that GPs should inform residents when they alter something in their medication. However, during the interviews, it became clear that this is currently not performed by every GP. All types of HCPs, GPs included, indicated that some GPs do not inform residents about medication changes. It was also deduced from the interviews that some GPs’ consultation routine made this practically impossible. Some visit the resident before consulting the resident’s medical file, which results in a potential medication change after seeing the resident. Others consult the resident’s medical file and discuss potential changes with the available staff and do not visit the resident afterwards. These routines imply that GPs do not formally inform the resident about their decision.

Subsequently, with regard to informing residents about medication changes, nurses put themselves in a second place, believing it is their task when the GP fails to do so. They seemed to take more responsibility when it comes to informal caregiver involvement in the medicines’ pathway. According to the nurses that were interviewed, it is their job to inform these caregivers about medication changes. Other HCPs, such as pharmacists and care aids, agreed with this fragmentation of responsibilities with regard to either informing residents and informal caregivers about medication changes or providing them with more medication-related responsibilities.

*“Initially, I would say the GP because he still makes the final decision and has the authority, which might be expressed too roughly. Patients [residents] will listen better to the GP than to the nurse or, in this case, the pharmacist who they have never seen before. So, I would say GPs and nurses. Pharmacists when they need to.”*

*Pharmacist 2, actively working for NH for 2 years*

It was deduced from the interviews that the fragmentation of responsibilities towards resident and informal caregiver involvement is rather based on assumptions and expectations towards one another’s duties. Although not recorded in any policy framework, assumptions and expectations of all types of HCPs were identical. Additionally, when one HCP fails to complete his assumed duties, another one seemed to take over. Therefore, it could be concluded that the fragmentation of HCPs’ responsibilities towards involvement in medication decision-making and the medicines’ pathway is based on unspoken but mutual agreement (see Fig. 1, ‘Features of resident and informal caregiver involvement’). Besides this, HCPs also expressed expectations towards residents and informal caregivers, indicating they expect them to ask questions or make remarks about the resident’s medication when they feel the need to.

### Healthcare professional’s perception of resident’s and informal caregiver’s desires and capabilities as a potential driver or barrier for resident and informal caregiver involvement

Most HCPs acknowledged potential benefits of involving residents and informal caregivers in the medicines’ pathway, but only to a limited extent. Perceived benefits included a higher sense of belonging for both residents and informal caregivers and the prevention of disagreements with families about installed medication changes (see Fig. 1, *‘Perceived consequences of involvement’*). However, their perception of residents’ and informal caregivers’ desire and capabilities to be involved was found to be an important factor in perceiving opportunities for their involvement (see Fig. 1, *‘Drivers and barriers for involvement’*). Most HCPs indicated to believe that residents nor informal caregivers have the desire for such thing, nor did they believe both groups have the cognitive capabilities to do so. Consequently, naming concrete opportunities to increase resident and informal caregiver involvement in medication decision-making and the medicines’ pathway did not occur. Likewise, HCPs failed to name the exploration of residents’ medication-related preferences or goals as an opportunity for their involvement.

*“There will be some [relatives] who want to be present [during the GPs consultation]. But they have never indicated to miss not being able to be present when the doctor visits their mother or father. I haven’t heard that yet.”*

*Nurse 5, actively working in NH for 5 years*

Two important reflections on this matter were noted during the interviews with HCPs within one NH, showing that the NH’s philosophy and the individual HCP’s attitude may transcend the perception of resident’s and informal caregiver’s desires and capabilities (see Fig. 1, *‘Drivers and barriers for involvement’*). Although agreeing with the potential lack of intellectual capabilities among some NH residents, these HCPs indicated that a lot of people would still be capable of learning new things, including the acquirement of new knowledge on medication-related topics. Additionally, it was emphasized that it is the responsibility of the HCPs to adapt their communication style and language to the resident’s or informal caregiver’s capabilities and not the other way around.

*“There is a large population of illiterate and uneducated people. Such conversations [about medication] are very challenging for those people. (…) Let alone that they will keep up with a conversation about medication management and risks thereof. We’ll be asking a lot of them. As such, I think that should be linked to an introduction on the topic. Cause those aren’t always people who are not able to learn anymore. They just never received that information.”*

*Director 2, actively working in NH for 6 years*

### Individual role perception of the healthcare professional as a driver or barrier for resident and informal caregiver involvement

Despite the more general aspects described above, differences in HCPs’ approaches of involvement of residents and informal caregivers were noticed. This, in turn, had an impact on their actions taken or opportunities perceived with regard to resident and informal caregiver involvement in the medicines’ pathway. A first contrast was noted between HCPs with an authorizing role and those with an executive role in residents’ daily medication use. It seemed as if HCPs with a more authorizing duty or role perception regarding residents’ medication named more examples of current actions and perceived involvement opportunities, opposed to those whose task or role perception consists of a more executive approach. This difference was seen, for example, between GPs (who clearly have a decisive vote in the decision-making process; i.e. authorizing duties), and pharmacists (who only play a limited role therein and are mainly responsible for the delivery of medication to the NH; i.e. executive duties). Besides this, inter-individual differences in role perception and actions were noticed within groups of HCPs, mainly within care aids and nurses. Some of these HCPs indicated to solely brief GPs about residents’ symptoms and behaviors when asked for it, besides preparing and administering medication based on GPs’ directives (i.e. executive role perception), while others seemed to take more responsibility with regard to medication decision-making, ensuring a more collaborative practice (i.e. authorizing role perception).

*“What I do sometimes, I go through the medication lists and see if things should be stopped. If so, I take a post-it and attach it to the inside of our cabinet. Because you don’t always know when the GP will visit. There’s also one GP who visits for 12 residents so that takes a while. So once in a while, I check the medication lists and attach a post-it to the cabinet [for the GP].”*

*Nurse 6, actively working in NH for 7 years*

Individual role perception could therefore be seen as either a driver or a barrier for naming opportunities for resident and informal caregiver involvement in medication decision-making and the medicines’ pathway (see Fig. 1, ‘Drivers and barriers for involvement’).

### Need for organizational control, qualitative and safe use of medication as barriers for increasing resident’s and informal caregiver’s autonomy

Healthcare professionals acknowledged the fact that residents live in the NH, which makes them strive towards an environment that evokes this ‘home feeling’. This was mentioned as a positive outcome of providing residents with more responsibilities with regard to their medication (e.g. to maintain a level of self-management, resulting in an increase of resident’s and informal caregiver’s autonomy) (see Fig. 1, *‘Perceived consequences of involvement’*).

*“We are trying to simulate a domestic environment. At home you can also go to the pharmacy for a box of Dafalgan [paracetamol] and keep this in your cupboard or not… But we can see perfectly what [medication] is being delivered. It could also be family that brings something. Fortunately, you need a prescription for most things so that’s not self-evident.”*

*Coordinating physician 1, actively working in NH for 25 years*

However, organizational concerns were noted that tempered this motivation. Firstly, the need of HCPs to maintain organizational control implicitly and explicitly influenced their perception of opportunities for resident and informal caregiver involvement (see Fig. 1, *‘Drivers and barriers for involvement’*). The increasing number of administrative procedures, imposed on NH staff, was found to strengthen this need among HCPs and was named as an impeding factor on the provision of holistic care, including resident and informal caregiver involvement. Additionally, the fear of resident and informal caregiver involvement causing organizational difficulties, such as less feasible and efficient work practices, resulted in a lack of perceived opportunities for resident and informal caregiver involvement in the medicines’ pathway (see Fig. 1, *‘Perceived consequences of involvement’*). Furthermore, HCPs expressed concerns about safety and quality of the resident’s medication use that could potentially be associated with higher resident and informal caregiver involvement (see Fig. 1, *‘Perceived consequences of involvement’*). They seemed to be anxious of residents making mistakes when provided with more responsibilities regarding their medication. Likewise, they seemed to fear a reduction of the quality of the resident’s treatment since they would not have full control over the storage of the medication, nor over the resident’s adherence, once residents would self-manage (some of) their medication.

*“It’s fighting against it, against [financial] profit [among other things]. They expect things from us that don’t really matter. All the information that needs to be registered. Everything needs to be checked. Make it a bit simpler again. (…) It’s always increasing and eventually, you have less time left for what really matters.”*

*Coordinating physician 4, actively working in NH for 25 years*

## Discussion

### Main findings

Actions of HCPs involving residents and informal caregivers in medication decision-making and the medicines’ pathway, mainly form a response to residents and informal caregivers expressing their interest in medication-related topics. This implies that HCPs do not proactively assess the information and participation needs of NHRs and informal caregivers. Nevertheless, HCPs provided multiple examples, showing that residents and informal caregivers self-initiate their involvement in medication-decision making and the medicines’ pathway. Therefore, it could be stated that information and participation needs are present among both groups. Furthermore, most of the HCPs’ actions are limited to informing residents and informal caregivers about medication changes, most often about those changes that could potentially result in observable consequences, simply to avoid disagreements. Initiatives by residents and informal caregivers on the other hand, seem to be more advanced and indicate a preference that is more extensive than the receipt of information about medication changes. Healthcare professionals provided examples that show a desire among residents and informal caregivers to be involved during the decision-making process, as well as a desire for more autonomy or independence with regard to the resident’s medication. Thus, our findings add to the existing literature that most HCPs are unaware of residents’ and informal caregivers’ information and participation needs with regard to medication-related decisions.

Moreover, the exploration of care preferences and goals of residents and informal caregivers was not mentioned as an opportunity by either one of the HCPs, although this is key to their involvement in decision-making and the provision of PCC [25, 26].

Clearly, HCPs’ actions are largely determined by their perception of residents’ and informal caregivers’ desire and capabilities to be involved in medication-decision making and the medicines’ pathway. The perception that NHRs do not want or are incapable to be involved in medical matters because of cognitive or physical limitations, has been described before [11], but has not been described with regard to medication-related activities. Healthcare professionals seem to have a general negative impression of residents’ and informal caregivers’ desire and capabilities and do not see that differences in residents’ and informal caregivers’ desires and capabilities may warrant an a priori patient-centered approach. Actively offering opportunities for involvement, ranging from the provision of information to being actively involved in decision-making, would acknowledge the existence of variation in preferences for involvement in decision-making between patients [10, 27, 28]. Overall, the opportunities should take into account communication difficulties that have previously been mentioned by older adults as an important barrier to being involved in medication decision-making [28]. Likewise, the need for communication support, tailored to the resident’s individual needs, should be tackled [29].

The idea that residents are not capable to be involved in the medicines’ pathway or parts of it, is also elicited in the reluctance of many HCPs to allow residents to self-manage their medication. Although this was acknowledged as a way to provide residents with a certain level of independence, it was clouded by the fear of a decrease in quality and safety of the resident’s medication use. A study by Maddigan et al. showed that cognitive limitations and higher medication regimen complexity are important predictors for lower self-management capacity among older adults, aspects that also characterize a significant part of NHRs and their treatment, but certainly not all. Additionally, the study showed that education and sufficient support might improve the adult’s capacity and can lead to a reduction of self-administration errors [30].

Healthcare professionals indicated that the main responsibility to involve residents in medication-related activities lies with the resident’s GP. With regard to involving informal caregivers, responsibility seems to shift towards the nurses who care for the resident. Nevertheless, these responsibilities are not specified in any policy document and are only based on a mutual but unspoken agreement between HCPs. The phenomenon of NH staff deriving responsibility to the resident’s GP to explore pharmaceutical goals of care has been described before [31]. Multiple explanations for this phenomenon were deduced from the interviews performed in our study. Residents’ and informal caregivers’ trust in the resident’s GP were identified as a first reason to have all types of HCPs name the GP as responsible to involve residents in medication-related activities, including decision-making. Nevertheless, previous research has shown that trust in GP may act as both a facilitator and a barrier towards involvement in medication decision-making [28]. Furthermore, the indispensable role of the resident’s GP in the medicines’ pathway was a second reason to name him responsible. Additionally, the individual role perception of HCPs seemed to have an influence on the perception of one’s own responsibilities towards involvement of residents and informal caregivers.

Based on these findings, we believe that a framework or policy document (which is currently non-existing) could help in clarifying the responsibilities of each HCP towards the involvement of residents and informal caregivers in medication decision-making and the medicines’ pathway. Likewise, it could be a starting to point to achieve structured involvement practices, keeping in mind the information and participation needs of individual residents and informal caregivers.

Actions of and perceived opportunities by HCPs were influenced by several organizational aspects. The NH’s philosophy was derived as one influencing factor in the realization of resident and informal caregiver involvement in medication decision-making and the medicines’ pathway. This aspect has been highlighted before by a scoping review on organization characteristics that influence the implementation of shared decision-making [32]. However, our findings show that solely having a philosophy with a general focus on resident and informal caregiver involvement is not enough and does not ensure their involvement in medication decision-making and the medicines’ pathway. The NH’s philosophy only acts as an influencing factor when the NH staff is being actively engaged and frequently reminded of this philosophy, highlighting the importance of supportive supervisory relationships [33]. Still, resident and informal caregiver involvement in medication-related activities is impeded by the need for organizational control. The phenomenon of the health system forming a restraint to resident involvement in decision-making has already been acknowledged [12, 26]. Likewise, HCPs feeling the need to maintain control over prescribing and medication administration activities to ensure safety, quality and continuity of care has also been previously described [34].

### Implications

Although PCC has been on the agenda for many years, our findings show that resident and informal caregiver involvement in medication decision-making and the medicines’ pathway remains limited in (Belgian) NHs. Therefore, initiatives, both on an individual and on an organizational level, to create and improve awareness among HCPs on the opportunities for and benefits of involvement in care are needed, aiming to show HCPs that resident and informal caregiver involvement can improve physical and mental well-being, satisfaction with care, and QoL [3, 35–38]. Likewise, efforts should be made to increase HCPs’ knowledge and know-how on how to provide PCC and involve residents and informal caregivers in medication-related activities, including how to elicit residents’ and informal caregivers’ medication-related goals and preferences. Furthermore, educational support is needed to heighten their awareness of the limitations placed by the healthcare system in order to help them to be proactive in their efforts to empower residents and informal caregivers.

### Strengths and limitations

Like any other study, this research has its strengths and limitations. An important strength is the inclusion of the expanded healthcare team in the interviews, from four different types of NHs. All types of HCPs involved in one or more medication-related activities in the NH were interviewed. Furthermore, the analysis of the interview data was performed by an interprofessional team, ensuring investigator triangulation and encouraging rigor and reliability of the findings.

The main limitation of this study is the limited number of interviews for each type of HCP, impeding data saturation on the level of each professional group.

## Conclusion

Resident and informal caregiver involvement in medication decision-making and the medicines’ pathway remains limited in nursing homes. Resident and informal caregiver involvement in medication decision-making and the medicines’ pathway is mainly initiated by residents and informal caregivers themselves. Although this indicates that both groups have information and participation needs, these were not fully acknowledged by HCPs. Furthermore, HCPs failed to name opportunities for resident and informal caregiver involvement in medication decision-making and the medicines’ pathway, which possibly explains why their current actions are mainly reactive and unstructured. Healthcare professionals’ actions are influenced by their perception of residents and informal caregivers’ desire and capabilities to be involved, as well as the perception of their own professional role. Moreover, HCPs’ initiatives are partly determined by a set of organizational factors, including the NH’s philosophy. As such, we can conclude that there is a need for initiatives, both on an individual and on an organizational level, to create and improve awareness on opportunities to improve resident and informal caregiver involvement in medication decision-making and the medicines’ pathway, and to develop concrete examples and tools to support this involvement.

## Data Availability

Transcripts analysed during the current study are available from the corresponding author on reasonable request. Transcripts are written in the language in which the interview was performed (i.e. Dutch or French).

## Abbreviations

CP: Coordinating physician
GP: General practitioner
HCP: Healthcare professional
NH: Nursing home
NHR: Nursing home resident
PCC: Person-centered care
RESPECT: RESident’s Participation in the Evaluation and Customization of Therapy
